# Pulmonary Cryptococcosis Mimicking Lung Cancer: A Diagnostic Challenge

**DOI:** 10.7759/cureus.47597

**Published:** 2023-10-24

**Authors:** Nishant Allena, Mrudula Kolli, Sudheer Tale, Pahel Soibam, Avishek Layek

**Affiliations:** 1 Department of Internal Medicine, BronxCare Health System, New York, USA; 2 Department of Internal Medicine, Gayatri Vidya Parishad Medical College, Visakhapatnam, IND; 3 Department of Internal Medicine, Maharajah's Institute of Medical Sciences, Visakhapatnam, IND; 4 Department of Pulmonary and Critical Care Medicine, Medicover Hospitals, Visakhapatnam, IND; 5 Department of Pulmonary and Critical Care Medicine, Jawaharlal Nehru Institute of Medical Sciences, Imphal, IND; 6 Department of Chest Medicine, All India Institute of Medical Sciences, Rishikesh, Rishikesh, IND

**Keywords:** ebus-guided fna, bronchoscopy, lung cancer, immunocompetent, pulmonary, cryptococcosis

## Abstract

Pulmonary cryptococcosis, although rare, maybe seen in both immunocompromised and immunocompetent patients. Cryptococcosis presenting as a lung mass mimicking lung cancer is very rare. Here, we report our experience with pulmonary cryptococcosis presenting as a lung mass mimicking malignancy in an immunocompetent patient. In this case, the patient presented to us with left-sided pleural effusion and lung mass on computed tomography (CT) of the chest. Bronchoscopy and endobronchial ultrasound (EBUS)-guided fine needle aspiration cytology (FNAC) was performed, which showed cryptococcal organisms. He responded well to oral anti-fungal therapy without any need for surgical interventions.

## Introduction

Cryptococcosis, also known as torulosis, is a subacute or chronic mycotic infection caused by *Cryptococcus neoformans*. Infection is acquired by the inhalation of aerosolized particles. The clinical presentation of cryptococcosis varies along a spectrum, from asymptomatic pulmonary colonization to severe pneumonia with respiratory failure and life-threatening meningitis [1]. Pulmonary cryptococcosis in immunocompromised patients can be severe and can be rapidly progressive, whereas in immunocompetent patients, it may be asymptomatic and usually remains confined to the lung as pulmonary nodules, although some may develop serious meningitis and disseminated infection [2]. There are only a few case reports of pulmonary cryptococcosis presenting as lung mass in immunocompetent patients.

## Case presentation

**Figure 1:**
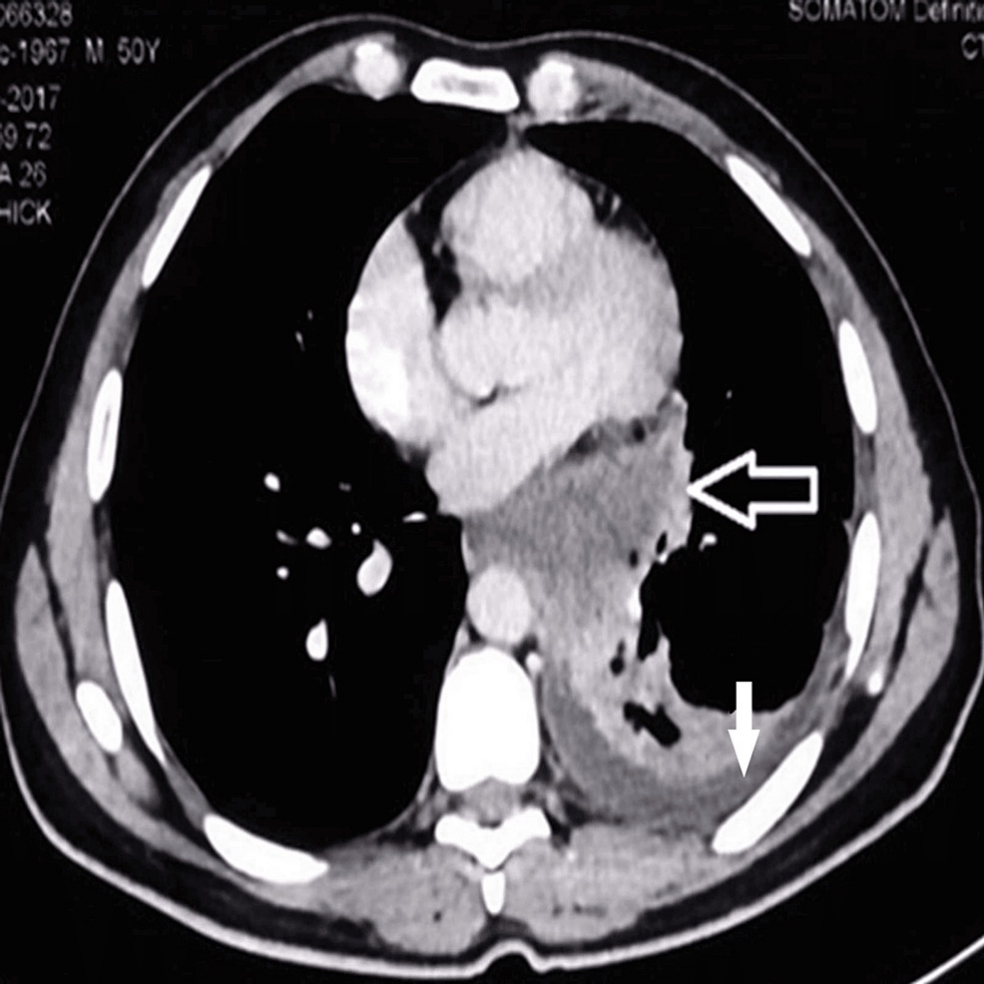
Computed tomography (CT) of the chest Black arrow showing a left hilar mass and white arrow showing ipsilateral effusion

A 50-year-old male confectioner with no history of diabetes mellitus, hypertension, or other comorbidities presented to our outpatient department with a six-month history of fever, cough with expectoration, and left-sided pleuritic chest pain associated with loss of weight and appetite. He was a non-smoker and non-alcoholic and had no history of high-risk behavior or exposure to birds. Initially treated with antibiotics by a local physician without much improvement, he was referred to our hospital for further management. On presentation, he had no neurological symptoms and was hemodynamically stable, afebrile, conscious, and oriented. Respiratory system examination revealed decreased breath sounds in the left infra-axillary and infra-scapular area, while the rest of the systemic examination was within normal limits. Initial investigations, such as complete blood counts, renal function tests, liver function tests, and blood sugars, were within normal limits except for leukocytosis with eosinophilia. Chest radiograph revealed a left-sided pleural effusion and left hilar prominence. Sputum examinations for tuberculosis (acid fast bacilli smear and GeneXpert) were negative. Pleural fluid analysis revealed lymphocytic predominant exudative effusion with low adenosine deaminase (24.8 U/L). Malignant cytology was negative. A contrast-enhanced computed tomography (CECT) of the chest showed an enhanced left hilar mass with ipsilateral pleural effusion (Figure 1).

**Figure 2:**
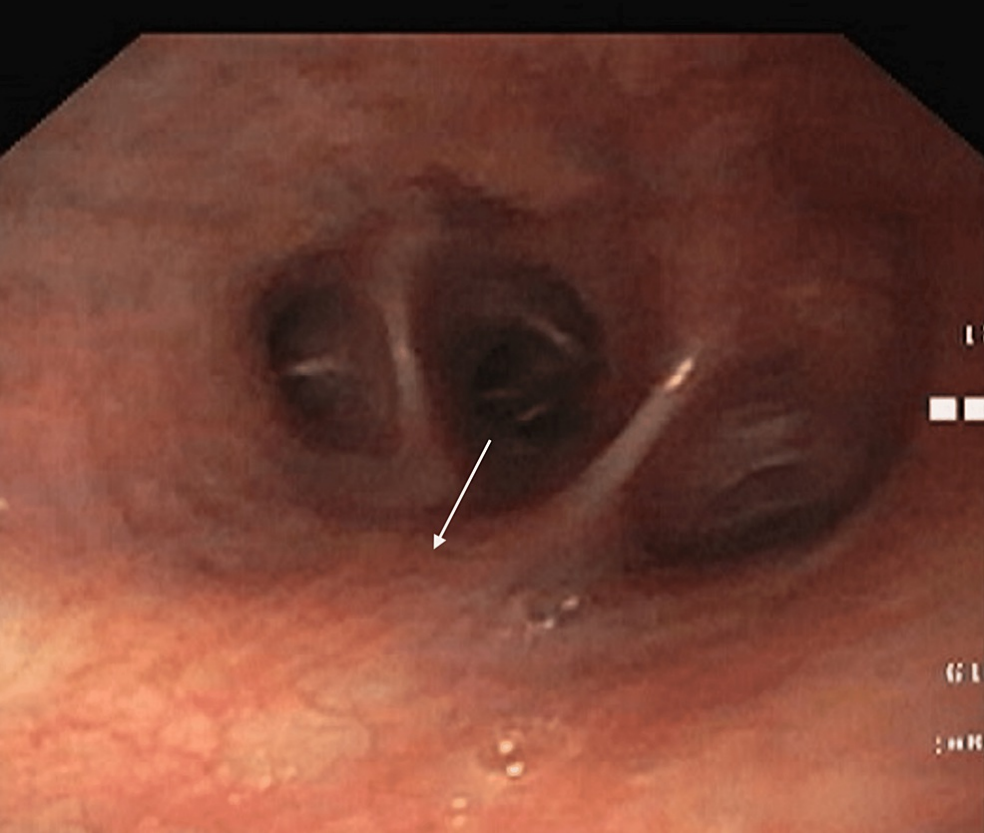
Bronchoscopy image of the left upper lobe White arrow showing mucosal erythema

A fiberoptic bronchoscopy was performed under local anesthesia, which showed mucosal erythema and mild mucoid secretions in the left tracheobronchial tree (Figure 2).

**Figure 3:**
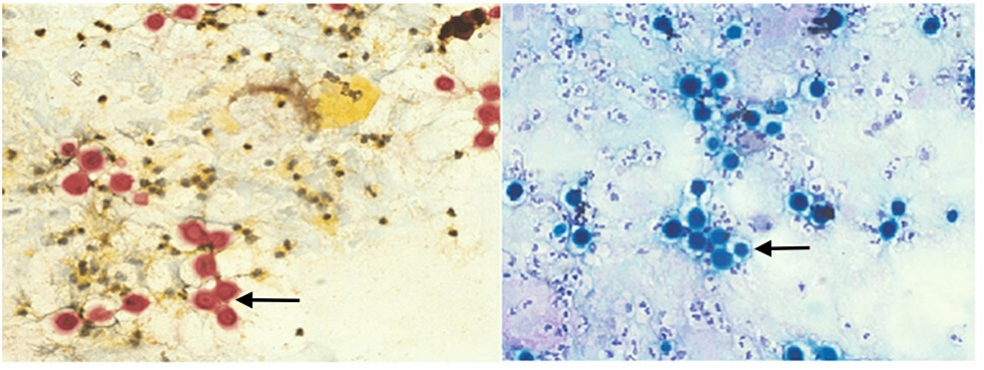
Mucicarmine and peroidic acid-Schiff (PAS) stain of bronchoalveolar lavage fluid Black arrows showing round to oval encapsulated yeast demonstrating *Cryptococcus*

Bronchial washings showed yeasts consistent with *Cryptococcus* (Figure 3), and subsequent cultures grew *Cryptococcus gattii*. The cryptococcal antigen was not tested in our patient due to very low clinical suspicion. Furthermore, the value of serological tests in the diagnosis of this condition is doubtful. His viral markers (human immunodeficiency virus (HIV), hepatitis B surface antigen (HBsAg), and anti-hepatitis C (HCV) antibodies) were negative, and the CD4 count was normal. Endobronchial ultrasound (EBUS)-guided transbronchial needle aspiration (TBNA) from the hilar mass revealed *Cryptococcus *organisms within the granulomas. The culture of the same showed *Cryptococcus neoformans var. gatii *infection. Cytology and cell block samples were negative for malignancy.

**Figure 4:**
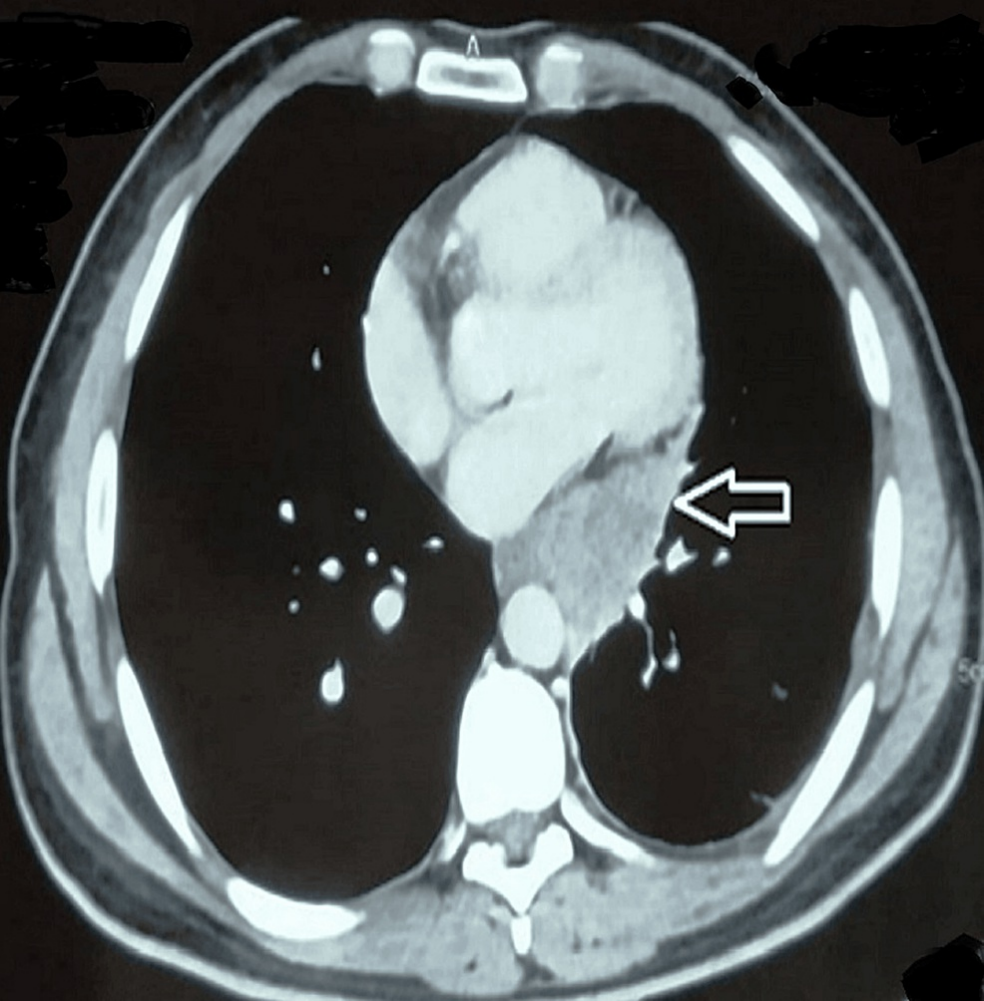
Follow-up computerized tomography (CT) of the chest Black arrow showing decrease in the size of the hilar mass and resolution of pleural effusion

The patient was initiated on oral fluconazole (400 mg/day) due to the mild-to-moderate nature of the disease. A repeat chest radiograph done after four weeks of treatment showed significant improvement of symptoms along with the resolution of pleural effusion. A repeat CECT of the chest (Figure 4) after five months of therapy showed complete resolution of pleural effusion with a decrease in the size of the mass. The patient was asymptomatic on regular follow-up, and the plan was to continue fluconazole for six more months along with monitoring of liver function tests.

## Discussion

Cryptococcosis is primarily a disease of immunocompromised individuals, especially those with HIV infection and transplant recipients. Other predisposing factors include type 2 diabetes mellitus, chronic liver and kidney disease, chronic steroid use, and defects of cell-mediated immunity. However, 10-40% of patients with cryptococcosis may not have any apparent predisposing factors [3]. The disease is caused by encapsulated yeast, *Cryptococcus*, which was previously considered as two biotypes of the same species (*Cryptococcus neoformans* var. *gatii* and *Cryptococcus* *neoformans *var. *neoformans*) but is now recognized as separate species, with *Cryptococcus neoformans* predominantly reported from immunocompromised patients, while* Cryptococcus gattii* infection has been associated with immunocompetent patients [4]. Inhalation of spores from environmental sources, such as pigeon droppings, rotten vegetation, and Eucalyptus trees, is the main source of infection.

Depending on the immune status of the individual, symptoms and radiological findings may vary, making the diagnosis of cryptococcosis extremely difficult [5]. Clinical manifestations can vary depending on the immune status of the individual. Cryptococcal meningitis is the most common manifestation in both immunocompetent and immunocompromised individuals. Pulmonary cryptococcosis is the second most common manifestation and is more common in immunocompetent patients [6].

The most common radiological findings of pulmonary cryptococcosis are single or multiple pulmonary nodules, masses mimicking lung cancer as seen in our case, air space consolidation, and patchy interstitial or alveolar infiltrates [7]. In our review of immunocompetent individuals with isolated pulmonary cryptococcosis masquerading as pulmonary masses, we found eight case reports treated with antifungal therapy and, in some cases, with lobectomy, as listed in Table 1.

**Table 1:** Brief summary of cases with pulmonary cryptococcosis masquerading as lung masses in immunocompetent individuals AMA: against medical advice; CXR: chest X-ray; RUL: right upper lobe; CT: computed tomography

Authors	Presentation	Imaging and investigations	Treatment	Culture	Outcome
Lu M, Raza R [8]	31-year-old male with cough	4.3 × 3.9 cm egg-shaped mass in the apical segment of the right lower lobe	Left AMA	No growth	Lost to follow-up
Guy JP, Raza S, Bondi E, Rosen Y, Kim DS, Berger BJ [9]	63-year-old Mediterranean-Caucasian woman presented with progressive dry cough	Multifocal pleural scarring, appearing metastatic in nature, throughout both lung fields with early mediastinal invasion in the right infrahilar region	Fluconazole 400 mg/day x 4 months	No growth	During the four-month follow-up, the chest radiograph showed a significant reduction of the basilar infiltrates bilaterally.
Haddad N, Cavallaro MC, Lopes MP, Fernandez JM, Laborda LS, Otoch JP, Ferreira CR [10]	17-year-old female presented with a 20-day history of chest pleuritic pain and fever	Nodular lesion with heterogeneous attenuation coefficient involving the bronchial tree in the left inferior lobe	Inferior lobectomy plus fluconazole	None	No follow-up mentioned
Barbosa AT, Colares FA, Gusmão Eda S, Barros AA, Cordeiro CG, Andrade MC [11]	69-year-old female with fever, chills, asthenia, jaundice, and cough with scant production of sputum	Heterogeneous mass in the left upper lobe, with air bronchogram adjacent to the mass	Fluconazole 300 mg/day for six months	C. neoformans	Partial regression of the lung mass, as seen on a second chest X-ray after six months
Mitchell DH, Sorrell TC [12]	41-year-old male with a two-month history of fever, sweats,nonproductive cough, and weight loss of 4 kg and pain in the tip of the right shoulder and weakness in the right arm and Horner syndrome on the right side of the face	CXR showing RUL mass	Lobectomy + 800 mg of amphotericin B (with 5-fluorocytosine) was given over a five-week period.	*C. neoformans *var *gatti*	Power and function in the right arm were almost normal, although Homer syndrome persisted after two years.
Kim YS, Lee IH, Kim HS, Jin SS, Lee JH, Kim SK, Song SH, Yoo J, Kim CH, Kwon SS [13]	74-year-old female with incidental finding on CXR	Chest computed tomography reveals a large consolidative mass in the left upper lobe (about 10 cm in the longest diameter) and multiple nodular consolidations with small nodules in both lungs.	Fluconazole 400 mg/day x 4 months	None	Chest radiographs shows significant decrease in the size of the consolidative pulmonary mass in the left upper lobe and multiple nodular consolidations with small nodules in both lungs.
Chang E, Wang AH, Lin C, et al. [14]	48-year-old male admitted with dyspnea	CT scan revealed a solitary right lung mass with invasion to the trachea	Oral itraconazole 400 mg/day for one year	C. neoformans	A CT scan of the thorax after two years showed no evidence of the pulmonary lesion.
Qiu S, Chen C, Li Y, et al. [15]	52-year-old male patient came to the hospital due to cough accompanied by cough with yellow phlegm, without fearless of cold, shivering, and high fever	18 mm irregular nodule in diameter distributed in the sub-pleural of the right lower lung	Itraconazole 200 mg po BID	None	No recurrence of lesion

Diagnosis of cryptococcosis depends on the site of infection, and it requires the demonstration of yeast cells in normal sterile tissues. Cultures from blood, sputum, and other body fluids are usually diagnostic [16]. In our case, we demonstrated yeast cells from bronchial washings using various staining techniques, such as mucicarmine, Giemsa's stain, and periodic acid Schiff (PAS). The culture showed growth of *Cryptococcus gattii*.

The management of pulmonary cryptococcosis depends on the immune status of the patient and the symptoms. The Infectious Disease Society of America guidelines recommend the use of fluconazole in mild to moderate pulmonary disease in immunocompetent patients and amphotercin B plus flucytosine in severe cases [17]. Therefore, in our case, the patient was treated with oral fluconazole and had significant symptomatic and radiological improvement.

## Conclusions

Our case underscores the significance of vigilance in recognizing pulmonary cryptococcosis as a potential culprit for lung masses that bear a resemblance to lung cancer, even in immunocompetent individuals. It is crucial to include this condition in the list of differential diagnoses for such lesions. Furthermore, it is imperative to administer prompt and robust medical intervention to avoid resorting to surgical measures. In light of these findings, it is evident that timely clinical, radiological, and histopathological evaluation plays a pivotal role in the successful management of pulmonary cryptococcosis.
